# Hepatocellular carcinoma progression during bridging before liver transplantation

**DOI:** 10.1093/bjsopen/zrab005

**Published:** 2021-04-11

**Authors:** P Renner, T Da Silva, A A Schnitzbauer, N Verloh, H J Schlitt, E K Geissler

**Affiliations:** Department of Surgery, University Medical Centre Regensburg, Regensburg, Germany; Department of Surgery, Robert-Bosch Hospital, Stuttgart, Germany; Department of Surgery, University Medical Centre Regensburg, Regensburg, Germany; Department for General, Visceral and Transplant Surgery, University Hospital Frankfurt, Goethe-University, Frankfurt, Germany; Department of Radiology, University Medical Centre Regensburg, Regensburg, Germany; Department of Surgery, University Medical Centre Regensburg, Regensburg, Germany; Department of Surgery, University Medical Centre Regensburg, Regensburg, Germany; Division of Personalized Tumor Therapy, Fraunhofer Institute for Experimental Medicine and Toxicology, Regensburg, Germany

## Abstract

**Background:**

Recipient selection for liver transplantation in hepatocellular carcinoma (HCC) is based primarily on criteria affecting the chance of long-term success. Here, the relationship between pretransplant bridging therapy and long-term survival was investigated in a subgroup analysis of the SiLVER Study.

**Methods:**

Response to bridging, as defined by comparison of imaging at the time of listing and post-transplant pathology report, was categorized into controlled *versus* progressive disease (more than 20 per cent tumour growth or development of new lesions).

**Results:**

Of 525 patients with HCC who had liver transplantation, 350 recipients underwent pretransplant bridging therapy. Tumour progression despite bridging was an independent risk factor affecting overall survival (hazard ratio 1.80; *P* = 0.005). For patients within the Milan criteria (MC) at listing, mean overall survival was longer for those with controlled *versus* progressive disease (6.8 *versus* 5.8 years; *P* < 0.001). Importantly, patients with HCCs outside the MC that were downsized to within the MC before liver transplantation had poor outcomes compared with patients who never exceeded the MC (mean overall survival 6.2 *versus* 6.6 years respectively; *P* = 0.030).

**Conclusion:**

Patients with HCCs within the MC that did not show tumour progression under locoregional therapy had the best outcomes after liver transplantation. Downstaging into the limits of the MC did not improve the probability of survival.

Prognostic factors determining the long-term success of liver transplantation in patients with hepatocellular carcinoma are still under discussion. A subgroup analysis of the SiLVER trial showed that disease control under bridging therapy is strongly associated with improved prognosis in terms of overall survival. However, in tumours exceeding the limits of the Milan criteria, downstaging did not restore the probability of survival compared with that of patients within the Milan criteria.

## Introduction

Tailored therapy based on individual patient and tumour factors is a goal of cancer management^1^. Hepatocellular carcinoma (HCC) is treated by different therapeutic means, but the benchmark algorithm is the Barcelona Clinic Liver Cancer system^2^^,^^3^. For HCC in liver transplant (LTx) recipients, guidance for patient selection is provided by the Milan criteria (MC), which define a subset of patients with favourable prognosis (single HCC lesions up to 5 cm in size *or* 1–3 lesions up to 3 cm, no extrahepatic manifestations, no macroscopic vascular invasion)^4^. Using patient eligibility based on the MC has substantially improved survival rates after LTx, producing results similar to those for patients with non-tumour indications^5^. However, categorizing patients with HCC solely based on tumour volume does not always result in accurate risk stratification for post-transplant disease-free (DFS) or overall (OS) survival. To address this issue, examining tumour growth and biology may reveal factors that more reliably predict HCC recurrence after LTx. Indeed, diagnostic tools to stratify post-LTx cancer risk at the time of HCC diagnosis are continuing to be refined^6^. For example, specific biomarkers (such as α-fetoprotein, AFP) may help to predict poor outcome in HCC^7^, but a reliable classification system based on such parameters is still lacking.

Apart from molecular or histopathological cancer features, simple cancer progression itself may represent a predictor of poor biological behaviour. In this regard, many patients with HCC on the waiting list for LTx undergo routine locoregional treat-and-wait testing, which may be regarded as an *in situ* functional test predicting the biological aggressiveness of each person’s cancer^8^. Previous studies have postulated that patients with HCC initially outside the MC that is downstaged successfully to within the MC have a prognosis similar to that of patients who were always within the MC^9^. Consequently, current United Network for Organ Sharing (UNOS) guidelines support a downstaging protocol (from outside to inside the MC), which focuses on response to bridging therapy, and an interval between bridging and transplantation^10^. Prospective data to validate this concept are lacking. The aim of the present study was to analyse bridging therapy in relation to tumour size and number on outcome after LTx.

## Methods

Data from the SiLVER study (Sirolimus in Liver Transplant Recipients with HCC study, NCT00355862) were used as a basis for this analysis^11^. The SiLVER study was a prospective randomized trial conducted in 13 European, Canadian, and Australian LTx centres, with the aim of testing whether mTOR inhibition could improve outcomes in patients receiving a LTx for HCC. For the purpose of the present subgroup analysis, only complete data sets from patients receiving locoregional HCC bridging treatment were included. Regarding HCC metrics, two sets of data were included in the analysis of each patient from the electronic case report system. The first set (pre-LTx) comprised the number and diameter of lesions determined by standard imaging techniques at the time of listing, and the second set (at the time of LTx) consisted of tumour data from the histopathology report obtained from the explanted liver. In accordance with Response Evaluation Criteria In Solid Tumours (RECIST)^12^, HCCs were defined as progressive if the sum of the tumour diameter(s) after LTx exceeded the pre-LTx sum by 20 per cent or more. HCCs with a change in tumour load of less than a 20 per cent increase at the time of LTx represented controlled disease.

### Statistical analysis

Continuous data are shown as mean(s.d.). The probability of survival was modelled by Kaplan–Meier analysis, with differences evaluated using the log rank test, and Cox regression analysis in SPSS version 25 (IBM, Armonk, New York, USA). Two-tailed *P* < 0.050 was considered statistically significant.

## Results

Of 525 patients in the SiLVER study, 350 underwent one or more HCC bridging treatments. Median follow-up was 5.3 (i.q.r. lower quartile: 2.4 years; upper quartile: 6.2 years) years. Bridging therapy consisted mainly of transarterial chemotherapy (47.1 per cent), radiofrequency ablation (19.1 per cent), and surgical resection (5.4 per cent) (*Table 1*). Mean(s.d.) age of the cohort was 57.8(7.0 years), and most of the study subjects were men (87.7 per cent). Mean time on the waiting list was 222 days, with a maximum of 7.25 years. Most tumours were smaller than 3 cm (58.3 per cent at time of listing), with the majority of the patients (51.7 per cent) presenting with single lesions. Approximately two-thirds of the patients had HCCs within the MC at the time of listing (68.3 per cent; detected by imaging) and transplantation (63.1 per cent; based on explant pathology report).

**Table 1:** Demographics, bridging therapy, and tumour data

	Listing (imaging)	Explant histology
**No. of patients**	350	
**Age (years)** ^*^	57.8(7.0)	
**Sex ratio (m : f)**	307 : 43	
**Time on waiting list (days)** ^*^	222(306)	
AFP before LTx (ng/ml)*^,†^	161(1125)	
**Bridging method**		
TACE	165 (47.1)	
RFA	67 (19.1)	
Resection	19 (5.4)	
Other	18 (5.2)	
Multiple	81 (23.1)	
**No. of nodules**		
0	0 (0)	61 (17.4)
1	181 (51.7)	134 (38.3)
2	69 (19.7)	60 (17.1)
3	49 (14.0)	42 (12.0)
4–5	41 (11.7)	28 (8.0)
> 5	10 (2.9)	25 (7.1)
**Size of largest nodule (cm)**		
No tumour	0 (0)	61 (17.4)
< 3.0	204 (58.3)	115 (32.9)
3.0–5.0	118 (33.7)	131 (37.4)
5.1–7.5	22 (6.3)	30 (8.6)
> 7.5	6 (1.7)	13 (3.7)
**Within Milan criteria**	239 (68.3)	221 (63.1)

Values in parentheses are percentages unless indicated otherwise;

* values are mean(s.d.).

† Data available for 349 patients. AFP, α-fetoprotein; LTx, liver transplantation; TACE, transcatheter arterial chemoembolization; RFA, radiofrequency ablation.

### Multivariable analysis of disease-free and overall survival

The variables age, time on the waiting list, AFP level before LTx, Milan status at listing (based on imaging), and tumour progression were included in a multivariable Cox regression analysis to determine their influence on DFS and OS in the bridging therapy subgroup. Although time on the waiting list and AFP level before LTx did not significantly affect survival, tumour progression was significantly associated with worse DFS (HR 1.50, 95 per cent c.i. 1.03 to 2.20; *P* = 0.035) and OS (HR 1.80,1.20 to 2.71; *P* = 0.005) (*Table 2*). Tumours outside the MC at listing were significantly associated with decreased DFS (HR 1.69, 1.15 to 2.47; *P* = 0.007), but only approached the threshold for significance regarding OS in this cohort.

**Table 2:** Results of multivariable Cox regression analysis

	Disease-free survival		Overall survival	
	Hazard ratio	*P*	Hazard ratio	*P*
**Age (per year)**	1.04 (1.01, 1.07)	0.011	1.05 (1.02, 1.08)	0.003
**Time on waiting list**	1.00 (1.00,1.00)	0.137	1.00 (1.00, 1.00)	0.213
**AFP before LTx**	1.00 (1.00, 1.00)	0.815	1.00 (1.00, 1.00)	0.809
**P rogression**	1.50 (1.03, 2.20)	0.035	1.80 (1.20, 2.71)	0.005
**Outside Milan at listing**	1.69 (1.15, 2.47)	0.007	1.45 (0.96, 2.19)	0.081

Values in parentheses are 95 per cent confidence intervals. AFP, α-fetoprotein; LTx, liver transplantation.

### Hepatocellular carcinoma progression and Milan criteria status

**Fig. 1:**
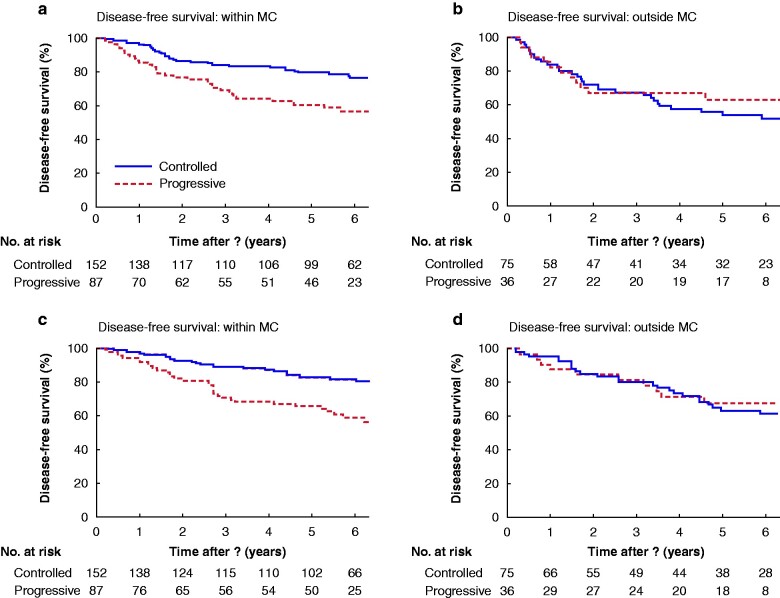
Kaplan–Meier estimates of disease-free and overall survival for patients with hepatocellular carcinoma within or outside the Milan criteria at listing based on imaging Disease-free survival for patients **a** within and **b** outside the Milan criteria (MC), and overall survival for patients **c** within and **d** outside the MC. **a** *P =* 0.002, **b** *P =* 0.490, **c** *P <* 0.001, **d** *P =* 0.726 (log rank test).

Tumour progression during bridging and the overall tumour load (MC status at listing) were separated into two subgroups for further survival modelling (*Fig. 1*). Mean DFS was 6.5 (95 per cent c.i. 6.1 to 6.9) years in the subgroup inside the MC with controlled tumour growth, compared with 5.5 (4.9 to 6.2) years in the subgroup with tumour progression (*P* = 0.002) (*Fig. 1a*). In the subgroup with disease outside the MC, no difference in DFS was noted between patients with controlled or progressive disease (*Fig. 1b*). Similar results were observed for OS. Patients initially inside the MC had significantly better mean OS when bridging resulted in no tumour progression (6.8 (6.4 to 7.1) years; 5-year survival 82.2 per cent) than in those with disease progression (5.8 (5.1 to 6.4) years; 5-year survival 65 per cent) (*P* < 0.001) (*Fig. 1c*). Response to bridging therapy in patients outside the MC did not affect OS; mean survival was 6.2 (5.5 to 7.0) years in patients with controlled disease *versus* 5.4 (4.6 to 6.2) years in those with progressive disease (*P* = 0.726) (*Fig. 1d*).

**Fig. 2:**
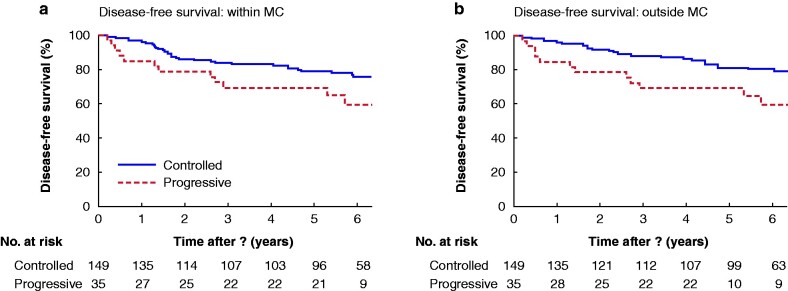
Effect of progression during bridging on disease-free and overall survival in patients with hepatocellular carcinoma within the Milan criteria both at the time of listing and at transplantation **a** Disease-free and **b** overall survival. **a** *P =* 0.089, **b** *P =* 0.021 (log rank test).

To analyse whether patients were affected negatively by tumour progression, even if the tumours stayed within the limits of imaging-based MC before LTx, 55 patients on bridging therapy who went from inside to outside the MC were excluded during the waiting period for LTx. In a subsequent analysis including only disease that stayed within the MC, patients with tumour progression during bridging had decreased DFS (mean 5.5 (4.5 to 6.5) *versus* 6.5 (6.1 to 6.9) years; *P* = 0.089) and significantly worse OS (5.5 (4.5 to 6.5) *versus* 6.8 (6.4 to 7.1) years; *P* = 0.021) than patients whose HCC was controlled during bridging therapy (*Fig. 2*).

### Downstaging in patients originally outside the Milan criteria

**Fig. 3:**
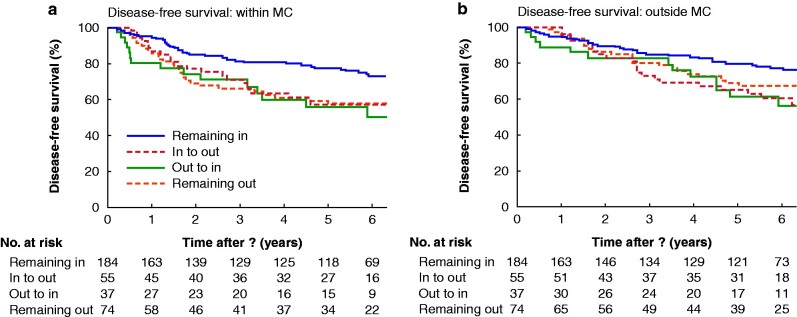
Kaplan–Meier model of disease-free and overall survival, according to behaviour of lesions during bridging/time on waiting list **a** Disease-free and **b** overall survival in relation to changes in Milan criteria status during therapy assessed by comparing listing imaging and explant histopathology. In, disease within limits of Milan criteria; out, disease outside limits of Milan criteria. **a** *P <* 0.050 (remaining in *versus* other subgroups; **b** *P =* 0.068 (remaining in *versus* remaining out), *P <* 0.050 (remaining in *versus* in-to-out and out-to-in subgroups) (log rank test).

Patients were categorized according to Milan status before and after bridging therapy (at listing (imaging) and at LTx (pathology)). In terms of DFS and OS, 184 patients who remained inside the MC throughout the LTx waiting period had significantly better outcomes than all other subgroups (*Fig. 3*). Thirty-seven patients with successful downstaging (out-to-in subgroup) had a mean DFS of 4.7 (95 per cent c.i. 3.7 to 5.6) years and OS of 6.2 (5.0 to 7.3) years, whereas patients who remained within the limits of the MC had superior survival, with mean DFS and OS of 6.3 (5.9 to 6.7) years (*P* = 0.008) and 6.6 (6.2 to 6.9) years (*P* = 0.030) respectively. The 5-year survival rate (OS) was 79.5 per cent in patients with HCC never exceeding the MC, compared with 61 per cent among those with disease downstaged from outside the MC.

## Discussion

As long as LTx is the only curative therapy for HCC in patients with cirrhosis, the question of patient selection will be an important matter for discussion^13^. The internationally applied (and often critiqued) MC allow definition of a subset of patients who benefit from LTx in terms of DFS and OS. Results from the present analysis of the SiLVER study are in line with predictions from the MC. However, the MC use a static tumour size/number evaluation and do not consider tumour progression during the LTx waiting period. The hypothesis related to this analysis was that tumour progression under the pressure of bridging therapy (ablate and wait^8^) before LTx may also be relevant to DFS and OS. Indeed, the present results showed that tumour progression is an independent risk factor for disease recurrence and mortality. Moreover, small (within the MC) but growing tumours that remained within the MC by the time of LTx were associated with outcomes similar to those for patients with disease outside the MC. This observation underscores the need for a comprehensive view of HCC lesions, including both growth dynamics and tumour load. Although a few small trials have suggested a predictive value of response to bridging before transplantation^14^^,^^15^, the SiLVER study provides a relatively large data set to demonstrate its association with DFS and OS, especially in the setting of small tumours. This is also consistent with a large US registry study^16^ that showed increasing AFP levels in patients undergoing locoregional therapy to be an indicator of relatively poor prognosis.

Another important aspect of the analysis was investigation of the effect of downstaging in patients with high-risk tumours (outside the MC before bridging). Some studies^12^^,^^17^ have suggested that reducing tumour volume with bridging therapy to within the limits of the MC allows LTx with a prognosis similar to that for patients within the MC. Recently, Kardashian and colleagues^18^ reported OS rates as high as 64.3 per cent in patients with downstaged tumours compared with 71.3 per cent for those within the MC, thereby underscoring the potential benefits of successful downstaging. In the SiLVER study cohort, downstaging from outside to within the MC resulted in distinctively worse outcomes than those for patients who remained within the MC, an observation shared by others^19^^,^^20^. Further refinement of selection criteria for patients with downstaged disease, for example by including AFP levels or waiting time^21^, may contribute to improved survival rates. However, without an RCT specifically addressing this issue, the present results suggest that considerable caution should be exercised when expecting that HCC downsizing will achieve expected outcomes.

The SiLVER study was neither powered nor initially designed to answer these questions, so limitations should be considered regarding the present analysis. First, bridging modalities were not standardized, and varied in both technique and frequency between study participants. Data acquisition was also limited by the fact that only consistently documented data for imaging at the time of diagnosis and in pathology reports from explanted livers post-LTx were available. Therefore, there was no planned interim analysis of tumour responses during bridging treatment, limiting knowledge about tumour kinetics and responses to therapy before LTx. Furthermore, patient enrolment into the SiLVER study was started in 2006, years before the introduction of the modified RECIST criteria, which would have allowed a more subtle analysis of tumour load and behaviour. This issue was addressed by introducing a simplified, but yet reliable categorization scheme (within *versus* outside the MC, controlled *versus* progressive tumour). It is also worth noting that the study protocol did not require (at that time) a waiting period between bridging and transplantation, as demanded by current UNOS criteria for patients with downstaged HCC.

Overall, this analysis has highlighted the potential negative prognostic impact of tumour progression in patients with HCC receiving locoregional therapy before LTx. The present results highlight the need for prospective trials that are specifically designed to assess HCC progression under pressure from bridging therapy, before making the critical decision regarding how to best to use organs for LTx.

## Notes

Members of the International SiLVER study group are co-authors of this study and are listed under the heading Collaborators.
